# Supplementary material for: Histologic Grade Is Predictive of Incidence of Epidermal Growth Factor Receptor Mutations in Metastatic Lung Adenocarcinoma
Source: Med Sci (Basel). 2017 Dec 11;5(4):34. doi: 10.3390/medsci5040034 (PMC5753663; doi:10.3390/medsci5040034)
Supplement: Supplementary file 1 [file medsci-05-00034-s001.pdf]

Supplemental materials
Levy et al.

Table S1. Frequency of EGFR mutation by age groups

Age Group	Number of positive cases by age group (N=83)	% of positive cases by age group versus total positive cases (N=83)	% of positive cases by age group versus all cases in age group (N=277)	p value
35-39 (N=3)	2	2.4	66.7	0.07
40-49 (N=18)	7	8.4	38.9	
50-59 (N=46)	19	22.9	41.3	
60-69 (N=87)	25	30.1	28.7	
70-79 (N=86)	17	20.5	19.8	
80-92 (N=37)	13	15.7	35.1	

Table S2. Distribution of EGFR mutations by histologic grade (one case harbored both exon 18 and 21 mutation)

Mutation Type	Histologic Grade					
EGFR Mutation	III N=11	II to III N=1	II N=34	I N=23	Unknown N=15	Total N=84
Exon 18 Mutation	0	0	1	2	1	4
Exon 19 Deletion	7	1	21	9	7	45
Exon 20 Duplication, Insertion, mutation	0	0	2	2	2	6
Exon 21 L858R Mutation	4	0	10	10	5	29
